# microRNA-25 targets PKCζ and protects osteoblastic cells from dexamethasone via activating AMPK signaling

**DOI:** 10.18632/oncotarget.13698

**Published:** 2016-11-29

**Authors:** Jian-bo Fan, Wei Liu, Xin-hui Zhu, Hong Yi, Sheng-yu Cui, Jian-ning Zhao, Zhi-ming Cui

**Affiliations:** ^1^ Department of Orthopaedics, The Second Affiliated Hospital of Nantong University, Nantong 226001, Jiangsu, PR China; ^2^ Department of Orthopaedics, Jinling Hospital, Nanjing Medical University, Nanjing 210008, Jiangsu, PR China

**Keywords:** Dexamethasone, Osteoblastic cells, microRNA-25, PKCζ, AMP-activated protein kinase (AMPK)

## Abstract

AMP-activated protein kinase (AMPK) activation could protect osteoblasts from dexamethasone (Dex). This study aims to provoke AMPK activation via microRNA downregulation of its negative regulator protein kinase C ζ (PKCζ). Results show that microRNA-25-5p (miR-25-5p) targets PKCζ's 3’ untranslated regions (UTRs). Forced-expression of miR-25 downregulated PKCζ and activated AMPK in human osteoblastic cells (OB-6 and hFOB1.19 lines), which thereafter protected cells from Dex. Reversely, expression of antagomiR-25, the miR-25 inhibitor, upregulated PKCζ and inhibited AMPK activation, exacerbating Dex damages. Notably, PKCζ shRNA knockdown similarly activated AMPK and protected osteoblastic cells from Dex. AMPK activation was required for miR-25-induced osteoblastic cell protection. AMPKα shRNA or dominant negative mutation almost completely blocked miR-25-induced cytoprotection against Dex. Further studies showed that miR-25 expression increased NADPH activity and suppressed Dex-induced oxidative stress in osteoblastic cells. Such effects by miR-25 were abolished with AMPKα knockdown or mutation. Significantly, miR-25-5p level was increased in patients’ necrotic femoral head tissues, which was correlated with PKCζ downregulation and AMPK hyper-activation. These results suggest that miR-25-5p targets PKCζ and protects osteoblastic cells from Dex possibly via activating AMPK signaling.

## INTRODUCTION

Long-term and/or excessive medication of dexamethasone (Dex) or other glucocorticoids (GC) will lead to different degrees of bone damages, including osteoporosis and osteonecrosis [1]. Osteoblast apoptosis was observed in the bones of GC-taking patients [1–3]. Dex could directly induce cytotoxic effect to osteoblasts, which may contribute to subsequent bone damages [4, 5]. Dex was added directly to cultured osteoblasts/osteoblastic cells to mimic GC-induced bone damages [4, 6–9]. Groups including ours are studying the pathological mechanisms of GC-induced osteoblast damages, and developing possible intervention strategies [8–13].

AMP-activated protein kinase (AMPK) is a serine/threonine protein kinase that is composed of a catalytic subunit (α) and two regulatory subunits (β and γ) [14–16]. AMPK functions as a sensor of cellular energy status via phosphorylating its downstream targets, *i.e*. acetyl-CoA carboxylase (ACC) [14–16]. Recent studies suggested that AMPK and it-regulated signalings can also promote cell survival [17]. Activated AMPK inhibits oxidant stress via activating nicotinamide adenine dinucleotide phosphate (NADPH) and inhibiting ATP depletion [18]. Further, AMPK could also activate cytoprotective autophagy to inhibit cell apoptosis [19, 20].

Recent research has explored the potential function of AMPK in osteoblasts/osteoblastic cells. It was shown that AMPK activation by Compound 13, a novel AMPK activator [21], protected osteoblasts from Dex [7]. Similarly, Zhu *et al*., showed that A-769662, another AMPK activator, protected osteoblasts from hydrogen peroxide (H_2_O_2_) [22]. Reversely, inhibition of AMPK, either genetically or pharmacologically, aggravated H_2_O_2_-induced osteoblast cell death [23]. The results of these studies imply that AMPK activation exerts pro-survival functions in osteoblasts/osteoblastic cells.

Existing evidences suggested that protein kinase C ζ (PKCζ) may act as a negative regulator of AMPK [24]. Activated PKCζ was shown to phosphorylate and inactivate LKB1, the AMPK kinase [24]. PKCζ inactivation, knockdown or mutation resulted in LKB1 de-phosphorylation and subsequent AMPK activation [24]. Based on the above information, it is proposed that PKCζ silence would activate AMPK and protect osteoblasts/osteoblastic cells from Dex. Through searching the miRNA database (TargetScan v7.1), microRNA-25-5p (“miR-25-5p”) is found to selectively targets PKCζ. Importantly, we show that miR-25 downregulates PKCζ and protects osteoblastic cells from Dex possibly via activating AMPK signaling.

## RESULTS

### microRNA-25 downregulates PKCζ and activates AMPK signaling in human osteoblastic cells

**Figure 1:**
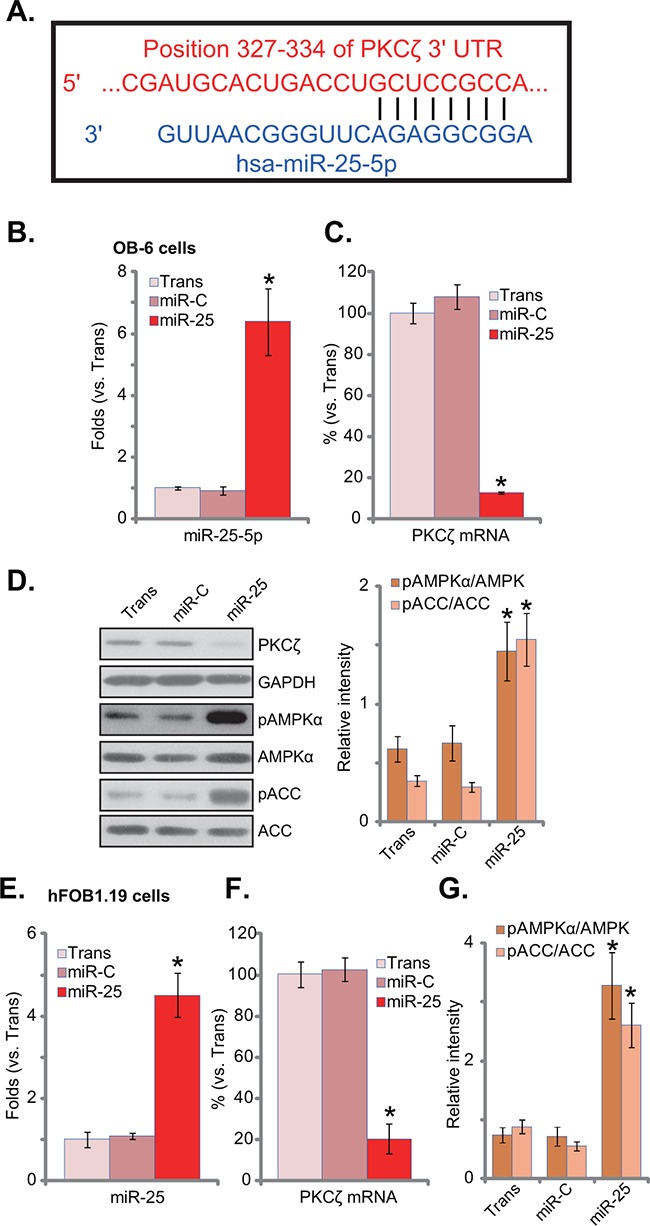
microRNA-25 downregulates PKCζ and activates AMPK signaling in human osteoblastic cells **A**. microRNA-25-5p (“miR-25-5p”) targets the 3’ untranslated regions of human PKCζ. Human osteoblastic OB-6 cells **B-D**. or hFOB1.19 cells **E-G**. were transfected with has-pre-miR-25 or the control microRNA (“miR-C”), and stable cells were established; Expressions of miR-25-5p (B and E) and PKCζ mRNA (C and F) were tested by quantitative real-time PCR (“qRT-PCR”) assay; Expression of listed proteins in these cells was tested by Western blot assay, and phosphorylations of AMPKα and ACCα were quantified (D and G). Experiments in this figure were repeated three times, and similar results were always obtained. “Trans” stands for transfection reagents only (B-G). *p<0.05 vs. group “miR-C” (B-G).

First, microRNA-25-5p (“miR-25-5p”) indeed targets PKCζ's 3’ untranslated regions (UTRs, position 327-334) (Figure 1A). To test if miR-25 could downregulate PKCζ in osteoblasts, a pre-miR-25-expressing construct was transfected it to OB-6 osteoblastic cells. Real-time PCR assay results in Figure 1B confirmed miR-25-5p over-expression in the stable OB-6 cells. miR-25 expression dramatically downregulated PKCζ mRNA (Figure 1C) and protein (Figure 1D) expression in OB-6 cells. Significantly, AMPK activation, tested by p-AMPK and p-ACC, was induced by miR-25 in OB-6 cells (Figure 1D). Similar results were also obtained in hFOB1.19 osteoblastic cells, where miR-25 expression (Figure 1E) downregulated PKCζ (Figure 1F), and activated AMPK signaling (Figure 1G). Notably, expression of miRNA control (“miR-C”) had no such effects (Figure 1B-1G). The expression of microRNA-25-3p (“miR-25-3p”) was also tested in these cells, and its level was not significantly increased following expression of pre-miR-25 (Supplementary Figure S1).

### microRNA-25 protects human osteoblastic cells from Dex

**Figure 2:**
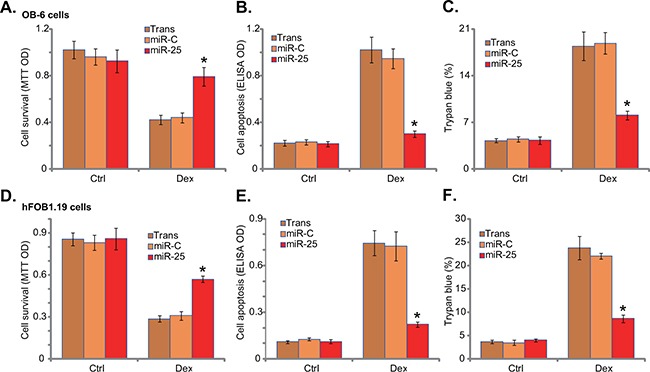
microRNA-25 protects human osteoblastic cells from Dex Stable osteoblastic OB-6 cells **A-C**. or hFOB1.19 cells **D-F**. expressing microRNA-25 (“miR-25”) or control microRNA (“miR-C”) were treated with or without Dex (1 μM) for 24 hours, cell viability (MTT assay, A and D), cell apoptosis (Histone DNA ELISA assay, B and E) and cell death (trypan blue assay, C and F) were tested. Experiments in this figure were repeated three times, and similar results were obtained. “Ctrl” stands for untreated control group. “Trans” stands for transfection reagents only. *p<0.05 vs. “miR-C” cells with Dex treatment.

Since AMPK activation would exert a pro-survival function in osteoblasts/osteoblastic cells [7, 22, 23], miR-25 expression should protect osteoblastic cells from Dex. As shown in Figure 2A, miR-25 expression indeed attenuated Dex-induced viability reduction in OB-6 cells. Meanwhile, Dex-induced OB-6 cell apoptosis (Figure 2B) and cell death (Figure 2C) were also significantly inhibited by miR-25. Similarly in hFOB1.19 cells, miR-25 alleviated cell viability reduction (Figure 2D), apoptosis induction (Figure 2E) and cell death (Figure 2F) by Dex. On the other hand, expression of miR-C showed no such activity (Figure 2A-2F).

### antagomiR-25 exacerbates Dex damages in human osteoblastic cells

**Figure 3:**
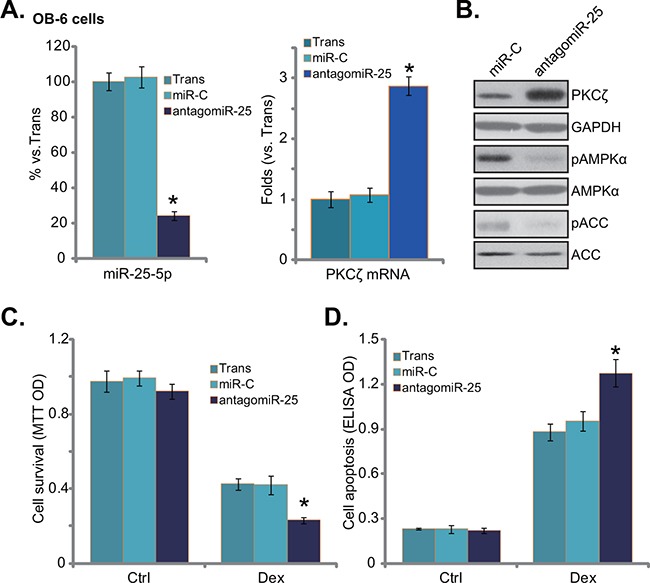
antagomiR-25 exacerbates Dex damages in human osteoblastic cells Expressions of miR-25-5p (**A**, left), PKCζ mRNA (**A**, right), and listed proteins **B**. in stable osteoblastic OB-6 cells with antagomiR-25 or non-sense control microRNA (“miR-C”) were shown; Above cells were treated with or without Dex (1 μM) for 24 hours, cell viability (MTT assay, **C**.) and apoptosis (Histone DNA ELISA assay, **D**.) were tested. Experiments in this figure were repeated three times, and similar results were obtained. “Ctrl” stands for untreated control group. “Trans” stands for transfection reagents only. *p<0.05 vs. “miR-C” cells.

To further confirm the role of miR-25 in osteoblastic cells, antagomiR-25, the miR-25 inhibitor, was introduced into OB-6 osteoblastic cells. Expression of antagomiR-25 did reduce miR-25-5p expression in OB-6 cells (Figure 3A, left). Reversely, PKCζ expression was upregulated (Figure 3A right, and 3B). Consequently, AMPK activation, or p-AMPK/p-ACC, was decreased in antagomiR-25-expressing cells (Figure 3B). Further studies showed that antagomiR-25 exacerbated Dex-induced OB-6 cell damages, leading to enhanced viability reduction (Figure 3C) and apoptosis activation (Figure 3D). Therefore, antagomiR-25 downregulates miR-25-5p and inhibits AMPK activation, which then potentiates Dex damages in human osteoblastic cells.

### PKCζ knockdown activates AMPK and protects human osteoblastic cells from dex

**Figure 4:**
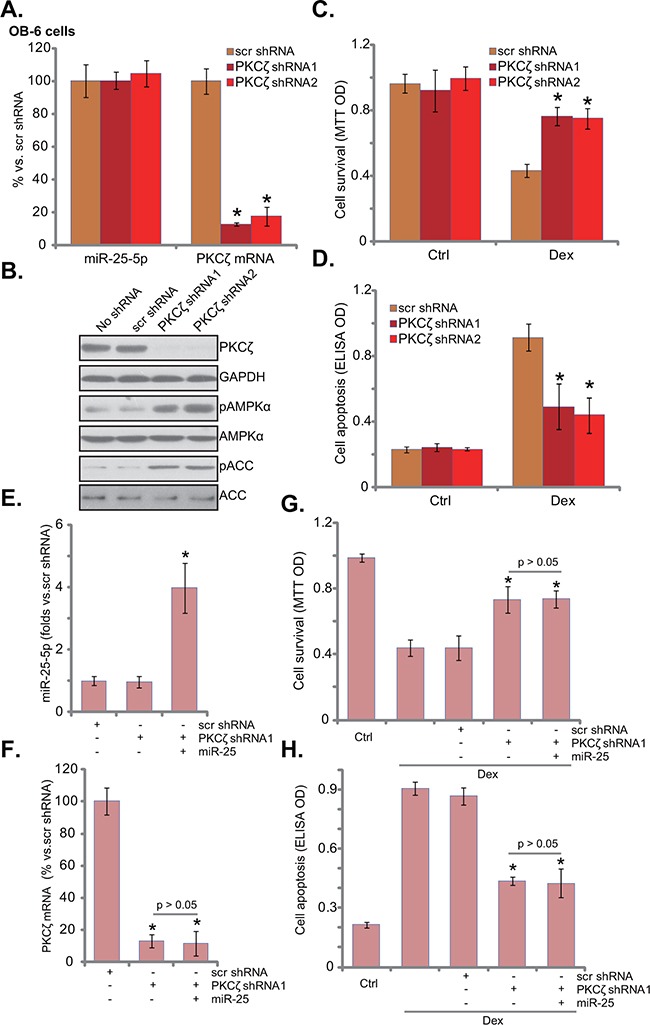
PKCζ knockdown activates AMPK and protects human osteoblastic cells from Dex OB-6 cells were infected with lentiviral PKCζ shRNA (“-1 or -2”) or non-sense control shRNA (“scr shRNA”), and stable cells were established; miR-25-5p and PKCζ mRNA expression levels in these cells were tested **A**., PKCζ protein expression and AMPK activation were also tested **B**. Above cells were treated with or without Dex (1 μM) for 24 hours, cell viability (**C**, MTT assay) and apoptosis (**D**, Histone DNA ELISA assay) were shown. PKCζ shRNA (“-1”)-expressing OB-6 cells were transfected with miR-25, expressions of miR-25-5p **E**. and PKCζ mRNA **F**. were shown; These cells were also treated with or without Dex (1 μM) for 24 hours, cell viability **G**. and apoptosis **H**. were tested. “Ctrl” stands for untreated control group. Experiments in this figure were repeated three times, and similar results were obtained. *p<0.05 vs. “scr shRNA” cells (A, C-H).

To test whether PKCζ downregulation was the reason of AMPK activation in miR-25-expressing osteoblastic cells, shRNA strategy was applied to knockdown PKCζ in OB-6 cells. Two non-overlapping PKCζ shRNAs (“-1/-2”) were applied, and each of them caused dramatic PKCζ downregulation (protein and mRNA) in OB-6 cells (Figure 4A and 4B). miR-25-5p level was unchanged with PKCζ knockdown (Figure 4A). Remarkably, PKCζ shRNA induced significant AMPK activation (p-AMPK/p-ACC) in OB-6 cells (Figure 4B), showing similar phonotype of miR-25 over-expression (Figure 1). More importantly, OB-6 cells with PKCζ silence were also protected from Dex, presenting with reduced viability reduction (Figure 4C) and apoptosis (Figure 4D).

miR-25 was then expressed in the PKCζ-silenced cells. qRT-PCR results confirmed miR-25-5p over-expression (Figure 4E) and PKCζ downregulation (Figure 4F) in these cells. Intriguingly, expression of miR-25 failed to further protect OB-6 cells from Dex in PKCζ-silenced cells (Figure 4G and 4H). In another words, miR-25 appeared in-effective against Dex when PKCζ was depleted (Figure 4G and 4H). These results imply that PKCζ might be the primary target of miR-25 in mediating its cytoprotective effect in osteoblastic cells.

### AMPK activation is required for miR-25-induced cytoprotection in osteoblastic cells

**Figure 5:**
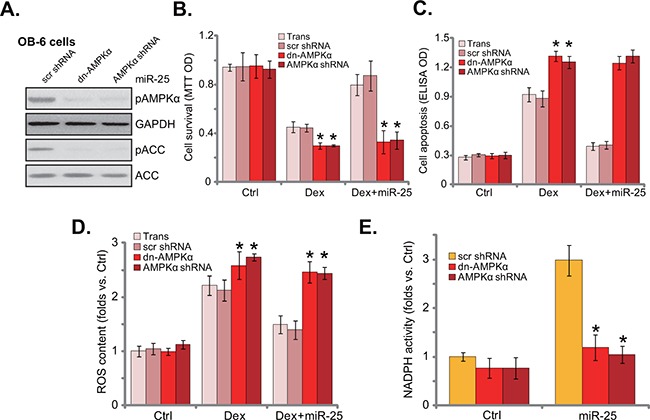
AMPK activation is required for miR-25-induced cytoprotection in osteoblastic cells Stable OB-6 cells with miR-25 were constructed with dominant negative AMPKα (“dn-AMPKα”, T172A), AMPKα shRNA, or the scramble control shRNA (“scr shRNA”), expressions of listed proteins in these cells were tested by Western blots **A**. Above cells were treated with or without Dex (1 μM), cell viability (MTT assay, 24 hours, **B**., apoptosis intensity (Histone DNA ELISA assay, 24 hours, **C**. and ROS content (DCFH-DA fluorescent dye assay, 6 hours, **D**. were tested; NADPH activity in above cells was also shown (4 hours, **E**). Experiments in this figure were repeated three times, and similar results were obtained. “Ctrl” stands for untreated control group. *p<0.05 vs. “scr shRNA” cells.

Thus, miR-25 expression activated AMPK and protected osteoblastic cells from Dex. The link between the two was then explored. Genetic strategies were utilized to block AMPK activation. AMPKα shRNA or dominant negative AMPKα (“dn-AMPKα”, T172A) was introduced to the miR-25-expressing OB-6 cells. Western blot results in Figure 5A demonstrated that miR-25-induced AMPK activation was almost blocked by AMPKα shRNA or mutation. Importantly, miR-25-induced cytoprotection against Dex was also abolished in AMPKα-silenced or -mutated cells (Figure 5B and 5C). Therefore, miR-25 was no longer cytoprotective when AMPK was silenced or mutated (Figure 5B and 5C). It should be noted that AMPKα shRNA or mutation augmented Dex-induced damages in OB-6 cells (no miR-25, Figure 5A-5C). These results are consistent with the antigomir-25 results (Figure 3), and further confirmed that AMPK inhibition would potentiate Dex-induced cytotoxicity in osteoblastic cells. Therefore, AMPK activation is required for miR-25-induced cytoprotection in osteoblastic cells.

Existing evidences have shown that AMPK activation could inhibit Dex-induced ROS production, therefore protecting osteoblasts/osteoblastic cells [7]. ROS production was also observed in Dex-treated OB-6 cells (Figure 5D). Significantly, miR-25 expression decreased Dex-induced ROS production in OB-6 cells. Remarkably, such anti-oxidant function by miR-25 was almost blocked with AMPK silence or mutation (Figure 5D). Since AMPK activation may exert anti-oxidant function via activating NADPH [7, 18, 23, 25]. The NADPH activity was then tested. Indeed, miR-25 increased NADPH activity in OB-6 cells (Figure 5E). AMPK shRNA knockdown or dominant negative mutation almost nullified miR-25-induced NADPH activation (Figure 5E). Based on the above results, we propose that miR-25 expression activates AMPK signaling to inhibit oxidative stress, and eventually protects osteoblastic cells from Dex.

### miR-25-5p upregulation correlates with PKCζ downregulation and AMPK activation in human osteonecrosis tissues

**Figure 6:**
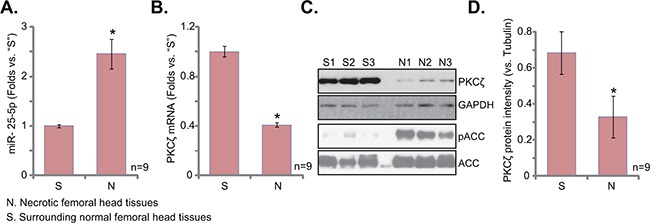
miR-25-5p upregulation correlates with PKCζ downregulation and AMPK activation in human osteonecrosis tissues Expressions of miR-25-5p **A**. and PKCζ mRNA **B**. in surgery-isolated femoral head tissues (both normal or “S” and necrotic or “N”) from GC-taking patients (n=9) were tested by qRT-PCR assay; Expression of listed protein was tested by Western blots (**C**, and PKCζ expression was quantified in **D**). *p<0.05 vs. “S” tissues.

It has been shown that AMPK activation was upregulated in patients’ necrotic femoral head tissues [23]. Here, miR-25-5p expression level in human necrotic femoral head tissues was tested, and its level was compared with that in the surrounding normal femoral head tissues (Figure 6A). In the necrotic tissues, the level of miR-25-5p was upregulated (Figure 6A), yet PKCζ (protein and mRNA) level was decreased (Figure 6B-6D). AMPK activity, indicated by p-ACC, was also increased in the necrotic femoral head tissues (Figure 6C). Therefore, miR-25 upregulation and PKCζ depletion could be at least one reason of AMPK activation in patients’ necrotic femoral head tissues [23].

## DISCUSSION

miR-25 is a member of miR-106b~25 cluster, which includes miR-106b, miR-93 and miR-25 [26]. In many cancers, miR-25 acts as an oncogene, and exerts pro-growth, anti-apoptotic, cell cycle-promoting activity via regulation of its target genes, including Bim, p57 and death receptor 4 (DR4) [27–29]. Its role in osteoblasts/osteoblastic cells has not been extensively studied. Here, miR-25 expression is pro-survival in human osteoblastic cells. Expression of miR-25 inhibited Dex-induced osteoblastic cell death and apoptosis. Reversely, antagomiR-25 intensified osteoblastic cell death by Dex.

Although miR-25-5p is a predicted target of PKCζ, it appears to be not conserved. As a matter of fact, the miRNA database (TargetScan v7.1) fails to find a single conserved miRNA for PKCζ. This could be due to the fact that the entire 3’UTR of PKCζ is poorly conserved among different cell types. Fortunately, here we show that miR-25-5p targets and downregulates PKCζ in osteoblastic cells, which activates AMPK signaling and protects cells against Dex. It will be interesting to test the potential effect of miR-25-5p on PKCζ and AMPK signaling in other cells.

It has been shown that Dex kills osteoblasts/osteoblastic cells via inducing oxidative stress [7, 30]. Reversibly, ROS inhibition could protect osteoblasts/osteoblastic cells from Dex [7]. Intriguingly, AMPK activation can exert anti-oxidant function and protect stressed cells [7, 18, 23, 31]. AMPK phosphorylates and inhibits ACC, thus reducing NADPH consumption [18]. Meanwhile, activated AMPK promotes NADPH synthesis though fatty-acid oxidation [18]. Here, miR-25 activated AMPK-NADPH signaling and inhibited Dex-induced ROS production. On the other hand, AMPK silence or mutation almost abolished miR-25-meidated anti-oxidant and cytoprotection in osteoblastic cells.

Excessive Dex usage could lead to osteoporosis [1, 2] and/or osteonecrosis [3]. In the Dex-taking patients’ bones, decreased number of osteoblasts and increased number of apoptotic osteoblast cells are often detected [1, 2]. Here, the *in vitro* results showed that miR-25-5p downregulated PKCζ and activated AMPK signaling to protect osteoblastic cell from Dex. Intriguingly, in human necrotic femoral head tissues, miR-25-5p expression was significantly increased, which was correlated with PKCζ downregulation and AMPK activation. Thus, in the following studies, it will be very interesting to further test the possible effect of miR-25-5p in animal model of Dex-induced bone damages.

In summary, these results conclude that miR-25 targets PKCζ and protects osteoblastic cells from Dex via activating AMPK signaling.

## MATERIALS AND METHODS

### Chemicals and reagents

Dex was obtained from Sigma Aldrich (Shanghai, China). All cell culture reagents were obtained from Gibco (Shanghai, China). All the antibodies were purchased from Santa Cruz Biotech (Santa Cruz, CA).

### Cell culture

The OB-6 [4] and hFOB1.19 [32] human osteoblastic cells were obtained from the Cell Bank of Shanghai Institute of Biological Science (Shanghai, China). Osteoblastic cells were cultured as described [4, 32].

### Quantitative real-time polymerase chain reaction (qRT-PCR) assay

As described in our previous studies [9, 11], total RNA was extracted by the SV total RNA purification system (Promega, Shanghai, China). Extracted RNA was reverse-transcribed through the reverse transcriptase (Promega). cDNA derived from 500 ng of RNA was amplified by quantitative real-time polymerase chain reaction (“qRT-PCR”). The SYBR Green PCR kit (Applied Biosystems, San Diego, CA) was applied to detect expression of targeted mRNAs. *GAPDH* primers (F-5′-AAG GTG AAG GTC GGA GTC-3′ and R-5′-TGT AGT TGA GGT CAA TGA AGG-3′) and *PKCζ* primers (F-5′-GCG TAC TGC GGC CAG TGC-3′ and R-5′-CTT GGC ATA GCT TCC ACG-3′) were described [33]. PCR was performed in triplicate and was conducted using a Real-Time PCR Detection System (7500; ABI, Shanghai, China). mRNA expression was quantified using the ^ΔΔ^Ct method using *GAPDH* as the internal control. Mature hsa-miR-25-5p expression was assessed using TaqMan microRNA assay using the primer described [34]. Mature hsa-miR-25-3p primer was described previously [35]. Five ng of total RNA was reverse-transcribed using TaqMan MicroRNA Reverse Transcription kit (Applied Biosystem) and the looped primer provided by the specific TaqMan microRNA assay [36]. All the primers and sequences were synthesized by Genepharm (Shanghai, China).

### miR-25/antagomiR-25 expression

Pre-miR-25, purchased from Applied Biosystem, was sub-cloned into pSuper-neo (OligoEngine, Seattle, WA) to generate miR-25 expression vector, which was transfected to the osteoblastic cells via Lipofectamine 2000 protocol (Invitrogen, Shanghai, China). Afterwards, cells were subjected to neomycin (1.0 μg/mL) selection for 10-12 days. Control cells were transfected with non-sense scramble microRNA-control (“miR-C”) (a gift from Dr. Lu's group [37]). The antagomiR-25 expression vector was described previously [38] and was transfected to osteoblastic cells with Lipofectamine 2000. Stable cells were established via selection. Mature miR-25 expression in the stable cells was tested by the qRT-PCR assay.

### Western blot assay

As described [9, 11], cell lysates were extracted via RIPA lysis buffer (Bio-sky, Nanjing, China). Aliquots of 30 μg lysates per sample were electro-transferred on 10% SDS-PAGE gel, following by transfer to PVDF membranes. The blots were then incubated with designated primary and secondary antibodies. The antigen-antibody binding was detected via enhanced chemiluminescence (ECL) reagents. ImageJ software was applied to quantify protein band.

### Cell death detection

Cell death was tested by counting cells using a cytometer after addition of trypan blue, which stained the cytoplasm of dead cells. Cell death percentage (%) = the number of trypan blue stained cells/the number of total cells (×100%) [11].

### Cell viability assay

Cell viability was measured via the routine 3-[4,5-dimethylthylthiazol-2-yl]-2,5 diphenyltetrazolium bromide (MTT) assay described in our previous studies [9, 11].

### Apoptosis assay by enzyme-linked immunosorbent assay (ELISA)

As described [39], the Histone-DNA Apoptosis ELISA Detection Kit (Roche, Palo Alto, CA) was applied to quantify cell apoptosis with indicated treatment.

### shRNA knock and stable cell selection

The two lentiviral shRNAs (GV248-puromycin vector) against human PKCζ were designed, synthesized and verified by Genepharm Co. (Shanghai, China). The AMPKα shRNA was described in our previous study [39]. Osteoblastic cells were seeded onto 6-well plates with 50% of confluence. The lentiviral shRNA (10 μL/mL) were added to cultured cells for 24 hours. Afterwards, cells were cultured in puromycin (1 μg/mL)-containing complete medium, until resistant colonies can be identified (10-14 days). The expression of target protein (PKCζ or AMPKα) in the stable cells was tested by Western blot assay. The scramble lentiviral shRNA (Santa Cruz) was added to the control osteoblastic cells.

### AMPK dominant negative mutation

The dominant-negative mutant of AMPKα (dn-AMPKα, T172A) construct was a gift from Dr. Lu's group [40], which was transfected to osteoblastic cells via Lipofectamine 2000 [40], and stable cells were selected via neomycin (1 μg/mL, Sigma). Transfection efficiency was always verified via Western blot assay in the stable cells.

### NADPH activity assay

NADPH activity assay was described in previous studies [23, 41]. Briefly, after treatment of cells, the lysates were incubated with NADP-cycling buffer plus glucose-6-phosphate dehydrogenase (G6PD, Sigma) at 60 °C for 30 min [23]. Afterwards, glucose 6-phosphate (G6P, Sigma) was added to the mixture, and the change in absorbance at 570 nm was measured every 30 s for 4 min at 30 °C. The concentration of NADP+ was calculated by subtracting [NADPH] from [total NADP]. NADPH activity was then calculated through NADPH/NADP+ [41].

### Reactive oxygen species (ROS) assay

ROS production was measured via a DCFH-DA fluorescent dye (Invitrogen). After treatment, cells were incubated with 1 μM of DCFH-DA (Invitrogen) for 30 min. Cells were then washed and analyzed for fluorescence using the flow cytometer (BD, Shanghai, China). The ROS intensity in the treatment group was normalized to that of control group.

### Human tissue specimens

Surgery-isolated fresh necrotic femoral head tissues and their surrounding normal femoral head tissues were collected from Dex-taking patients. Fresh tissue specimens were dissolved in tissue lysis buffer (BiYunTian Biotechnology Research Institute, Nantong, China) and were subjected to qRT-PCR assay and Western blot assay. The experiment protocols requiring human samples were approved by the institutional review board and ethics committee of all authors’ institutions, and written informed consent was obtained from each patient. A total of 9 patients were included. All studies were conducted according to the principles expressed in the Declaration of Helsinki.

### Statistics

The data presented were mean ± standard deviation (SD). Statistical differences were analyzed by one-way ANOVA followed by multiple comparisons performed with post hoc Bonferroni test (SPSS version 18.0). Values of *p* < 0.05 were considered statistically significant.

## SUPPLEMENTARY MATERIALS FIGURES


