# Supplementary material for: microRNA-25 targets PKCζ and protects osteoblastic cells from dexamethasone via activating AMPK signaling
Source: Oncotarget. 2016 Nov 29;8(2):3226–36. doi: 10.18632/oncotarget.13698 (PMC5356877; doi:10.18632/oncotarget.13698)
Supplement: Supplementary file 1 [file oncotarget-08-3226-s001.pdf]

microRNA-25 targets PKCζ and protects osteoblastic cells from dexamethasone via activating AMPK signaling

SUPPLEMENTARY FIGURE

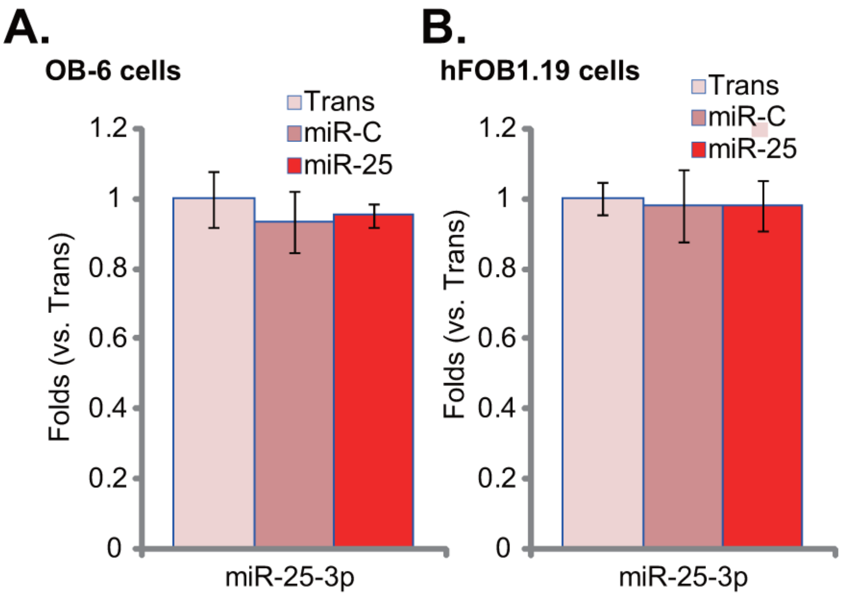

Supplementary Figure S1: Human osteoblastic OB-6 cells A. or hFOB1.19 cells **B.** were transfected with has-pre-miR-25 or the control microRNA ("miR-C"), and stable cells were established; Relative expressions of miR-25-3p was tested by quantitative real-time PCR ("qRT-PCR") assay; "Trans" stands for transfection reagents only. Experiments in this figure were repeated three times, and similar results were always obtained.
